# Association of Plasma Phospholipids with Age-Related Cognitive Impairment: Results from a Cross-Sectional Study

**DOI:** 10.3390/nu13072185

**Published:** 2021-06-25

**Authors:** Ting-Ting Liu, Shao-Jie Pang, Shan-Shan Jia, Qing-Qing Man, Yu-Qian Li, Shuang Song, Jian Zhang

**Affiliations:** 1 Chinese Center for Disease Control and Prevention, National Institute for Nutrition and Health, Beijing 100050, China; yuzhongting20@163.com (T.-T.L.); jssky.good@163.com (S.-S.J.); qqm0327@163.com (Q.-Q.M.); cnu_lyq@126.com (Y.-Q.L.); zhjian6708@aliyun.com (J.Z.); 2 Institute of Grain Quality and Nutrition Research, Academy of National Food and Strategic Reserves Administration, Beijing 100037, China; psj@ags.ac.cn

**Keywords:** phospholipid, cognitive impairment, plasma

## Abstract

Decreased concentration of phospholipids were observed in brain tissue from individuals with dementia compared with controls, indicating phospholipids might be a key variable in development of age-related cognitive impairment. The reflection of these phospholipid changes in blood might provide both reference for diagnosis/monitoring and potential targets for intervention through peripheral circulation. Using a full-scale targeted phospholipidomic approach, 229 molecular species of plasma phospholipid were identified and quantified among 626 senile residents; the association of plasma phospholipids with MoCA score was also comprehensively discussed. Significant association was confirmed between phospholipid matrix and MoCA score by a distance-based linear model. Additionally, the network analysis further observed that two modules containing PEs were positively associated with MoCA score, and one module containing LPLs had a trend of negative correlation with MoCA score. Furthermore, 23 phospholipid molecular species were found to be significantly associated with MoCA score independent of fasting glucose, lipidemia, lipoproteins, inflammatory variables and homocysteine. Thus, the decreased levels of pPEs containing LC-PUFA and the augmented levels of LPLs were the most prominent plasma phospholipid changes correlated with the cognitive decline, while alterations in plasma PC, PS and SM levels accompanying cognitive decline might be due to variation of lipidemia and inflammatory levels.

## 1. Introduction

Age-related cognitive impairment, characterized clinically by gradual progressive intellectual deterioration, and pathologically by neurofibrillary tangles, senile plaques, neuropil threads or neuron loss, is becoming a serious problem among aging societies around the world [1,2]. The pathophysiological changes associated with cognitive impairment were found to begin years before the emergence of clinical symptoms. Although numerous hypotheses have been advanced, the precise pathological processes underlying these adverse neurocognitive changes are not well understood [3]. Thus, identification of metabolic changes associated with cognitive decline is critical for both understanding the underlying pathogenesis and early diagnosis/intervention of cognitive impairment.

Phospholipid is a complicated lipid family containing phosphorus. Diverse permutations of head groups, backbones and fatty acyl chains generate hundreds of phospholipid molecular species in biosamples, generally classified as phosphatidylcholine (PC), phosphatidylethanolamines (PE), phosphatidylserine (PS), phosphatidylinositol (PI), phosphatidylglycerol (PG), phosphatidic acid (PA), sphingomyelin (SM) and their lyso-type (Lysophospholipids, LPLs). Accounting for a quarter of brain’s dry mass, phospholipids not only provide structural and functional variety for membrane of neural cells [4,5], but also participate in conduction of neurotransmitter and electrical signals [6].

Numerous studies on post-mortem brain samples reported decreased concentration of phospholipids in brain tissue from individuals with dementia compared to controls. The reduction of PI and PE levels in dementia brain is consistent in most studies, while decreased or unchanged PC and SM levels were observed in different studies [7,8]. The deficiency of plasmenyl phosphatidylethanolamine (pPE, plasmalogen sub-class of PE) in white matter achieved 40% at very early stage of dementia, while deficiency of pPE in gray matter increased from 10% to 30% during disease progression [9]. Meanwhile, a 73% decrease in plasmenyl phosphatidylcholine (pPC, plasmalogen sub-class of PC) were observed in the prefrontal cortex of dementia patients [10]. In addition, the general increase of PC and SM concentration in cerebrospinal fluid (CSF) was found positively associated with amyloid positivity and tau levels among cognitive impairment patients, and at the same time associated with progression from mild cognitive impairment to dementia in long-term follow-up [11,12]. Therefore, phospholipid might be a key variable in development of age-related cognitive impairment. The reflection of these neuro phospholipid changes in blood might provide both reference for routine diagnosis/monitoring of cognitive impairment and potential targets for intervention through peripheral circulation.

However, precise identification and quantification of phospholipid profile in plasma, including classes, sub-classes, and individual molecular species, are always challenging. Limited by phospholipid characterization techniques, most publications only focused on association of cognitive impairment and a few classes of plasma phospholipid, like only total pPE [13] or only plasma PC and SM [11,14]. At the same time, the conclusions obtained by the few studies with full-scale phospholipid profile are also inconsistent. Han et al. reported significant decreased level of SM in plasma of dementia patients [15], and Chatterjee et al. reported correlation between plasma PC/pPE species and brain amyloid load [16]. The divergence of these conclusions might be due to the small sample size (*n* < 60) on the one hand, and the cross-interference of diet or other metabolic processes on the other.

In this study, using liquid chromatography (LC) coupled with mass-spectrometry (MS), a total of 229 molecular species of plasma phospholipid were identified and quantified among 626 aged residents from three communities. Additionally, the association of plasma phospholipids with cognitive score was discussed comprehensively in this cross-section study. Other metabolic factors that may interfere with plasma phospholipid concentration and cognitive score were considered and controlled stepwise to find a direct link between plasma phospholipids and neurodegenerative processes.

## 2. Materials and Methods

### 2.1. Study Participants and Design

Residents aged 60 to 80 years were volunteered from three communities (Yuxing, Qingyuan and Jianxin community) located in Shijiazhuang, China. According to the medical record system, individuals with serious sequelae from cardiovascular and cerebrovascular diseases, diagnosed with mental disorder and clinical history of severe head trauma or encephalopathy were excluded. Individuals using drugs that regulate lipid metabolism and individuals declaring supplementation of phospholipids/omega-3 lipids were also excluded. A total of 626 residents were eventually enrolled as participants in this study.

For all participants, demographic information, cognitive assessment and fasting blood samples were collected. Plasma phospholipid profile and biochemical parameters related with lipid metabolism and cognitive impairment were further analyzed in laboratory. According to the principles of the Declaration of Helsinki, the study protocol was approved by the ethics committee of National Institute of Nutrition and Health, Chinese Center for Disease Control and Prevention (No. 2013-013, 4 March 2013). Written informed consent was gathered from all participants prior to investigation.

### 2.2. Demographic Variables

Basic demographic information was obtained according to a questionnaire, including gender, age, ethnicity and educational attainment.

### 2.3. Cognitive Assessment

The Montreal Cognitive Assessment (MoCA) was used for evaluation of cognitive function. The MoCA test includes 52 items that cover 8 cognitive domains (visuospatial/executive ability, naming, memory, attention, language, abstraction, delayed recall and orientation). The scores of MoCA test range from 0 to 30 in present study, and lower scores signify more impairment.

### 2.4. Plasma Collection and Storage

All the participants fasted overnight (10–14 h) before blood sampling to eliminate the short-term influence of diet on plasma phospholipids. Peripheral venous blood samples were collected using EDTA-K_2_ blood tubes (BD, Franklin Lakes, USA) and centrifuged immediately at 800× *g* for 30 min at 4 °C. The upper layer of plasma was divided and transferred into sterile tubes and stored at −80 °C before analysis.

### 2.5. Analysis of Biochemical Parameters

Biochemical parameters related to lipid metabolism or cognitive impairment were analyzed, including fasting glucose (Glu), total cholesterol (TC), triglycerides (TG), low-density lipoprotein cholesterol (LDL-C), high-density lipoprotein cholesterol (HDL-C), lipoprotein(a) (Lp(a)), apolipoprotein A1 (Apo A1), apolipoprotein B (Apo B), apolipoprotein E (Apo E), C-reactive protein (CRP), tumor necrosis factor-α (TNF-α), interleukin 6 (IL-6) and homocysteine (Hcy). TNF-α and IL-6 were analyzed by radioimmunity assay using a Wizard^2^ gamma counter (PerkinElmer, Billerica, USA). The other parameters were analyzed by enzymatic (Glu, TC, TG, LDL-C, HDL-C and Hcy) or immunoturbidimetry (Lp(a), Apo A1, Apo B, Apo E and CRP) method using a Hitachi 7600 automatic biochemical analyzer (Hitachi, Tokyo, Japan).

### 2.6. Analysis of Plasma Phospholipid Profile

#### 2.6.1. Extraction of Total Phospholipid

Total phospholipid was extracted from human plasma according to a modified Folch method. Briefly, 100 μL plasma and 10 μL deuterium labeled internal standards solution and 1.0 mL chloroform/methanol (2:1, *v*/*v*) were subjected to vortex mixing for 30 min. Then, 0.2 mL NaCl solution (0.9%) was added to the mixture. The resultant mixture was mixed for 30 min and centrifuged at 8000× *g* for 10 min. The chloroform layer was transferred into a new tube and dried under a gentle stream of nitrogen. All the samples were redissolved in 100 μL of chloroform/methanol (2:1, *v*/*v*) before analyses.

#### 2.6.2. Qualitative Analysis of Phospholipid Molecular Species in Plasma

According to a qualitative analysis method described in our previous publication [17], the phospholipid molecular species were identified in a mixed plasma sample (prepared by taken 10 μL from each plasma sample and mixed together) using a hydrophilic liquid chromatography-electrospray ionization-ion trap-time of flight-mass spectrometry (HILIC-ESI-IT-TOF-MS) system. Scan data of MS, MS^2^ and MS^3^ were collected under ESI^−^ mode. The phospholipids were preliminarily identified by LIPID MAPS Structure Database (LMSD). Detailed structures of individual molecular species were further confirmed by MS^2^ and MS^3^ spectra based on the fragmentation laws of phospholipids that described in our previous publication.

#### 2.6.3. Quantitative Analysis of Phospholipid Molecular Species in Plasma

The identified phospholipid molecular species were quantified according to a hydrophilic liquid chromatography-electrospray ionization-triquadrupole-mass spectrometry (HILIC-ESI-MS/MS) method as described and validated in our previous research [18]. The MS/MS data were collected in targeted MRM mode for all the identified phospholipid molecular species. The plasma samples were analyzed in random order, and blank samples were added in the sequence after every 10 samples to ensure no significant carryover of lipids, respectively. Individual molecular species of phospholipid were quantified using internal standard method (the standards used for quantification as shown in Table S1). A total of 229 phospholipids with detection rate higher than 50% in 626 plasma samples, including 62 PCs, 55 PEs, 30 SMs, 22 PSs, 19 PIs, 5 PGs, 3 Pas, 22 LPCs and 11 LPEs, were included in current study (as listed in Table S2).

### 2.7. Statistical Analysis

The undetected concentrations were assigned a value as half of the detection limit, and all the phospholipid molecular concentrations were log transformed for further statistical analysis. Distance-based linear model (DistLM), a permutation-based version of the multivariate analysis of variance, was employed to test the association of phospholipid matrix with MoCA score and biochemical parameters using Primer-e software (version 7.0, Quest Research Limited, Auckland, New Zealand). DistLM was performed by Bray–Curtis distance, with stepwise regression as selection procedure and 999 as the number of permutations. Weighted gene co-expression network analysis (WGCNA, R, version 1.63, Vienna, Austria) was used to determine modules of highly interconnected phospholipids. Neighborhoods of interconnected phospholipids were defined by topological overlap measure (TOM). The modules were represented by the first principal component of the metabolites included in the modules. Network plotting was performed using Cytoscape (version 3.8.2, National Institute of General Medical Sciences, Bethesda, MD, USA). Spearman’s correlation coefficients (*r_s_*) were calculated between MoCA score and phospholipid modules or individual phospholipid molecular species, using SAS software (version 9.4, SAS Institute, Inc., Cary, NC, USA). Multivariate regression of phospholipid molecular species and MoCA score were analyzed by orthogonal projections to latent structures (OPLS) model using SIMCA software (version 14.1, MKS Umetrics, Sweden). UV scaling was used for data pretreatment in the OPLS model.

## 3. Results

### 3.1. General Characteristics of Participants

In present study, a total of 626 participants were included, the median age was 64 years (range from 60 to 80), of which 230 (36.7%) were male, 617 (98.6%) were Han Chinese, and 481 (76.8%) had a primary or above education level (Table 1). The median score of MoCA test among participants was 25 (range from 8–30). The variables related with lipid metabolism includes fasting glucose, lipidemia and lipoproteins. The median value of Glu, TG, HDL-C, Lp(a), and Apo E was 5.3 mmol/L, 1.2 mmol/L, 1.2 mmol/L, 110.0 mg/L and 1.5 g/L, respectively; and the mean value of TC, LDL-C, Apo A1, and Apo B was 5.2 mmol/L, 3.1 mmol/L, 1.5 g/L and 4.0 mg/dL, respectively. Meanwhile, the cognitive impairment-related variables, including inflammatory variables (CRP, TNF-α, IL-6) and Hcy, were described in Table 1. The median value of CRP, TNF-α and IL-6 was 0.9 mg/L, 10.2 pmol/L and 102.5 pg/mL, respectively, and the median value of Hcy was 16.1 μmol/L.

### 3.2. Characteristics of Plasma Phospholipids

A total of 229 phospholipid molecular species were identified in participants’ plasma, including 62 PCs, 55 PEs, 30 SMs, 22 PSs, 19 PIs, 5 PGs, 3 PAs, 22 LPCs and 11 LPEs. The detailed concentration of the identified plasma phospholipid molecular species among participants was listed in Table S2. Additionally, the concentration of phospholipid classes was listed in Table 2. The median value of total phospholipid concentration in plasma was 2046.7 mg/L. PC was the most abundant class of plasma phospholipid in terms of both content and number of molecular species. PE had the second abundant number of molecular species but the fourth highest content among all the classes. Both PC and PE contain plasmanyl/plasmenyl sub-class. The plasmanyl PC/PE has one fatty acyl group connected to glycerin by ether bond, and the plasmenyl PC/PE (also known as plasmalogen) has one fatty acyl group connected to glycerin by vinyl ether bond. The proportion of pPE in PE (17.0%) is much higher than the proportion of pPC in PC (4.9%). SM, LPC and PI had the second, third and fifth abundant concentration, respectively. Although 22 PSs were detected in plasma, the total content of PS is only 1.6 mg/L, accounting for less than 1‰ of total phospholipid. As for PG and PA, only trace species and levels were detected.

### 3.3. Association of Phospholipid Matrix with MoCA Score and Related Biochemical Parameters

A distance-based linear model (DistLM) was conducted to assess the significance of association between the plasma phospholipid matrix and MoCA Score/biochemical parameters. Gender, ethnicity, age and education level were forced into the model as control. MoCA, Glu, TC, TG, LDL-C, HDL-C, Lp(a), Apo A, Apo B, Apo E, CRP, TMF-α, IL-6 and Hcy were included in the model in sequence for trial. As shown in Table 3, MoCA was strongly associated with phospholipid matrix with *p* < 0.001. Lipidemia variables and most lipoproteins were significantly associated with phospholipid matrix as well, including TC, TG, LDL-C, HDL-C, Apo A1, Apo B and Apo E. However, the R^2^ value is less than 0.02 when LDL-C, HDL-C, Apo A1, Apo B and Apo E were included into the model, indicating that the proportion of the variation in the phospholipid matrix explained by these variables is limited. Meanwhile, no significant association was observed between phospholipid matrix and Glu, Lp(a), Hcy or the inflammatory variables (CRP, TMF-α and IL-6).

### 3.4. Association of Phospholipid Clusters with MoCA Score

**Figure 1:**
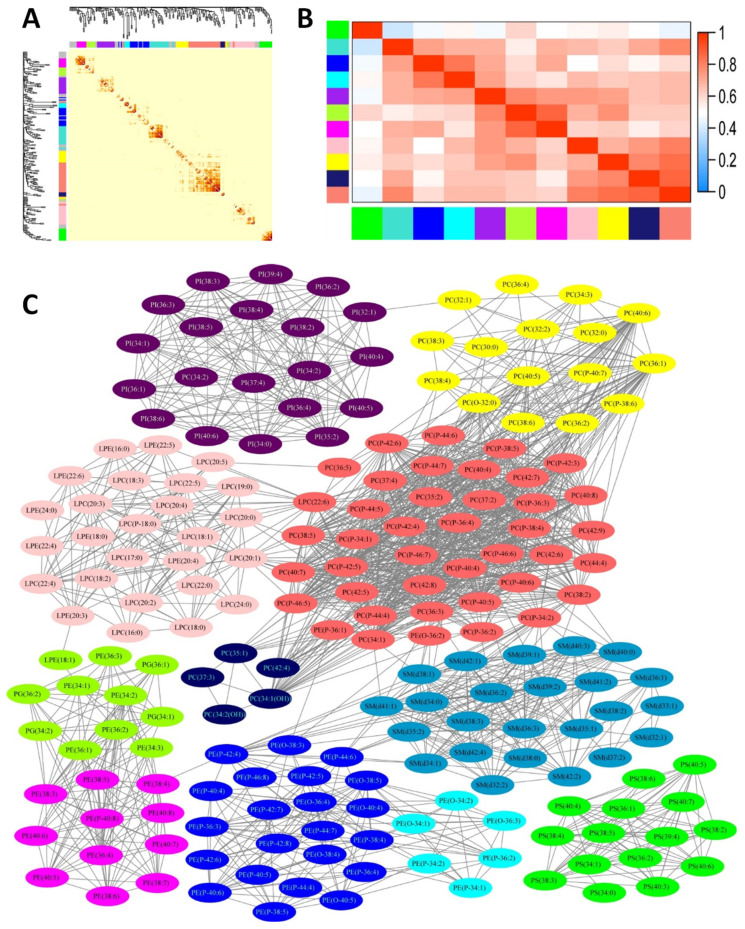
Weighted gene co-expression network analysis (WGCNA) of phospholipid profile. (**A**) Cluster dendrogram and heatmap correlation of 229 phospholipid molecular species. Color bars on the left and at the top represent 11 identified modules. Phospholipids that could not be clustered to any module are labeled gray. (**B**) Correlation heatmap among the 11 modules. Color bars on the left and at the bottom represent modules, and grid squares indicate Spearman’s correlation coefficients among module eigengenes. (**C**) Colors indicate the modules detected by topological overlap measure (blue and cyan: plasmanyl/plasmenyl PEs; magenta: diacyl PEs; greenyellow: diacyl PEs and PGs; salmon, yellow and midnightblue: PCs; green: PSs; purple: PIs; turquoise: SMs; pink: LPCs and LPEs).

The 229 phospholipid molecular species identified in plasma were clustered by WGCNA analysis. A total of 11 modules were generated. As shown in Figure 1, blue and cyan modules were composed of plasmanyl/plasmenyl PEs; magenta module was composed of diacyl PEs; greenyellow module was mainly composed of PGs and diacyl PEs; salmon, yellow and midnightblue modules was mainly composed of PCs; green module was composed of PSs; purple module was composed of PIs; turquoise module was composed of SMs; and pink module was composed of LPCs and LPEs. The detailed attribution of phospholipid molecular species among these modules were listed in Table S2. The association of module eigengenes with MoCA Score was investigated by Spearman’s rank correlation with control of gender, ethnicity, age, and education level. As listed in Table 4, the positive correlation between blue/magenta module and MoCA score was significant (*p* < 0.05), and the correlation coefficient between blue module and MoCA score is relatively higher. Besides, a trend of negative correlation of pink module and MoCA score was observed as well (*p* = 0.06).

### 3.5. Association of Individual Phospholipid Molecular Species and MoCA Score

**Figure 2:**
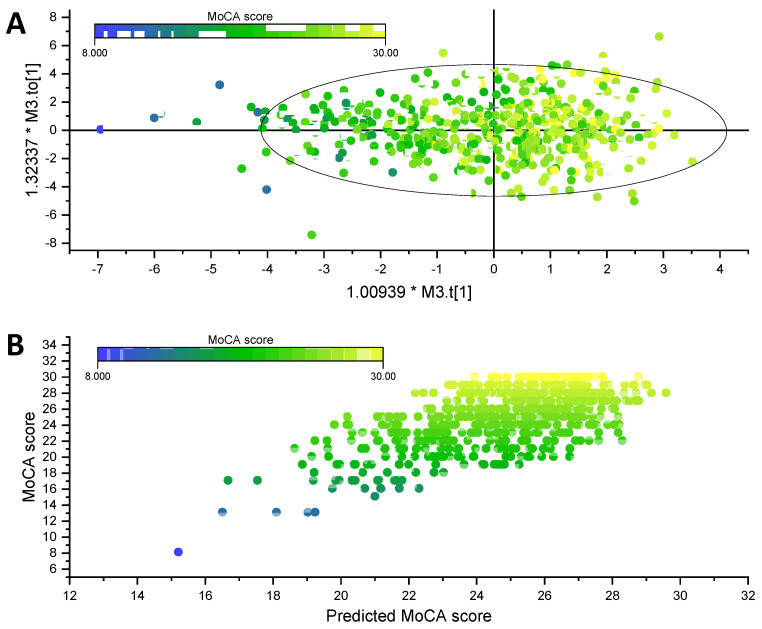
(**A**) Score plot of orthogonal projections to latent structures (OPLS) model constructed by gender, ethnicity, age, education and 23 phospholipid molecular species. (**B**) Regression of determined and predicted MoCA score by OPLS model.

The association of individual phospholipid molecular species and MoCA score was investigated by Spearman’s rank correlation with stepwise control of related variables. As demonstrated in Table 5, 45 phospholipid molecular species, including 22 PEs, 4 PCs, 10 SMs, 4 PSs, 1 PI, 1 LPE and 3 LPC, were significantly correlated with MoCA score with control of gender, ethnicity, age and education level. Of note, 18 of the 22 PEs were pPE with long-chain poly unsaturated fatty acid connected (LC-PUFA) on *sn*-2 position. However, seven PEs, three PCs and six SMs were no longer correlated with MoCA score significantly when lipidemia and lipoprotein variables were added as covariate (Model 2 and Model 3). Additionally, further controlling for inflammatory variables abolished the significant association of MoCA score with three SMs and three PSs (Model 4), while the controlling of Hcy did not further change the association of MoCA score with phospholipid molecular species (Model 5). Thus, the correlation between MoCA and 15 PEs (13 pPEs), 1 PC, 1 SM, 1 PS, 1 PI, 1 LPE and 3 LPCs was independent of Glu, lipidemia, lipoproteins, inflammatory variables and Hcy. With gender, ethnicity, age, education and these 23 phospholipid potential biomarkers, a multivariate regression was conducted by OPLS model. One predictive and one orthogonal component were consisted in the OPLS model with coefficient of determination R^2^Y = 0.41, prediction ability Q^2^ = 0.37, and CV-ANOVA significance *p* < 0.001. As shown in Figure 2, a graded distribution is observed based on MoCA score in the score plot.

## 4. Discussion

By applying an accurate targeted phospholipidomic approach, 229 phospholipid molecular species were characterized in plasma samples from 626 senile residents. According to our knowledge, this should be the first study using a large sample to evaluate the association of full-scale phospholipid profile with cognitive decline in the Chinese population. Significant association was confirmed between phospholipid matrix and MoCA score in distance-based linear model, and the network analysis further observed collective effects: two modules containing PEs were positively associated with MoCA score (with the stronger association in the module containing pPEs) and one module containing LPLs (LPCs and LPEs) had a trend of negative correlation with MoCA score. After stepwise controlling of related risk factors (including Glu, lipidemia, lipoprotein, inflammatory and Hcy), a total of 23 phospholipid molecular species, including 15 PEs (13 pPEs), 1 PC, 1 SM, 1 PS, 1 PI, 1 LPE and 3 LPCs, were found to be significantly associated with MoCA score.

According to the above results, the decrease in levels of pPEs containing LC-PUFA is the most prominent plasma phospholipid change accompanying cognitive decline in this study. Similar results were reported in several previous studies focusing only on pPE. By characterizing eight pPE molecular species, level of pPE(38:6) and pPE(40:6) were found significantly reduced in serum of patients with late-onset Alzheimer’s disease (LOAD) compared with control [19,20], and LOAD patients with pPE deficiency in serum performed significantly cognitive decline after one year [13]. Our results indicate that the reduced levels of 12 pPE molecular species may be associated with cognitive decline far prior to the development of dementia, since the MoCA scale used in this study is more sensitive to detect cognitive impairment in early stage compared with the Mini-Mental State Examination (MMSE) used in previous studies. Moreover, the association of pPE biosynthesis value (the combination of three pPE molecular species) with cognition was reported to be independent of Apo E in a US population [21], while the positive correlation between pPE levels and cognitive score was found to be independent of not only lipoproteins, but also fasting glucose, lipidemia, inflammatory variables and homocysteine in present study. The depletion of pPE observed in peripheral circulation is consistent with that observed in post-mortem brain tissue of LOAD patients [9,22]. Therefore, the systemic deficiency of pPE is considered to play an important role in the etiology of age-related cognitive impairment.

Thus far, the biological functions of pPEs, especially in the central nervous system (CNS), have not been fully unraveled. However, many clues have been derived from the pathology of pPE-deficient mice and men. As a special sub-class of PE, pPEs are particularly concentrated in brain and heart, accounting for more than 60% of PE in neurons [19]. Peroxisomes are involved in the initial steps of vinyl-ether bond synthesis and completion of pPE synthesis occurs in endoplasmic reticulum, which is very different from the synthesis of diacyl PEs. The congenital disorder of peroxisome accompanies the lack of pPEs and results lethal disease like Zellweger’s syndrome. The severe patients could hardly develop any cognitive abilities [23]. The deficiency of pPE in age-related cognitive decline might occur via two mechanisms: (1) impaired generalized function of peroxisome with dysregulation of alkyldihydroxyacetonephosphate synthase (AGPS), the key enzyme for synthesis of vinyl-ether bond [24]; (2) increased activity of plasmalogen-selective phospholipase A_2_ (PLA_2_), a key enzyme involved in the turnover and degradation of pPE with 30-fold specificity for pPEs versus diacyl PEs [25,26]. The pPEs are important components of cellular membranes and may be involved in the organization of cholesterol-rich membrane regions known as detergent-resistant microdomains, which have been implicated in cellular signaling and neurotransmitter vesicular fusion [27]. Additionally, the pPEs are speculated to be radical scavenger in CNS, since the vinyl ether has low dissociation energy and high sensitivity to oxygen free radicals. The myelin of pPE-deficient mice was significantly more vulnerable to ROS, and pPE augmentation reverses striatal dopamine loss in mice brain induced by oxidative stress [28,29]. Furthermore, it is speculated that pPEs participate in inflammatory regulation through the PUFA connected on *sn*-2 position. Insufficient pPEs in mice brain could activate microglia and up-regulate the expression of inflammatory factors, and extrinsic pPE containing LC-PUFA attenuate the LPS or Aβ_42_ induced inflammatory activation.

In addition, the augmented levels of LPE(16:0), LPC(16:1), LPC(22:0) and LPC(24:0) were also an important correlate of cognitive decline independent of fasting glucose, lipidemia, lipoproteins, inflammatory variables and homocysteine according to present study. Nevertheless, the relationship between LPL levels and cognitive function is inconsistence in various studies. Higher concentration of LPE(16:0), LPE(18:1) and LPG(18:1) was observed in plasma of mild LOAD patients compared with control [30]. On the contrary, Mapstone et al. reported that LPC(18:2) was depleted in the plasma of participants converted form normal cognition to mild cognitive impairment (MCI) or dementia compared with control group [31]. However, the concentration of LPC(18:2) was not associated with prevalence of MCI or dementia according to a cross-sectional study focused on the phospholipids identified by Mapstone et al. [32]. Moreover, a non-targeted metabolomics study reported decrement of LPC(16:0), LPC(18:0), LPC(18:1), LPC(18:2) and LPC(20:4) level in plasma of LOAD patients compared with control [33]. Thus, it appears that the relationship of plasma LPL levels and cognition is still confusing, which might be due to the diverse characterization method, population and sample size used in different studies. Abundant evidence exists regarding the capacity of free LPC to increase cytosolic Ca^2+^ and activate inflammatory signaling pathways [34], which supports the negative correlation between LPL and cognitive function observed in our study to a certain extent. However, the precise pathological role of LPLs in cognitive decline remains to be defined.

Beside pPE and LPL changes, alterations in plasma PC and SM levels accompanying cognitive decline were also reported in previous studies. The decreased levels of seven plasma PC molecular species were found to be correlated with the higher risk of conversion from normal cognition to mild cognitive impairment (MCI)/dementia, of which only PC(40:2) and PC(36:6) were associated with prevalence of MCI and dementia in cross-section study [31,32]. Decrements in plasma total SM and eight SM molecular species were associated with memory impairment in cross-sectional analyses, while high level of total SM predicted cognitive impairment longitudinally [15,35]. In present study, it is interesting that 4 PC and 10 SM molecular species were correlated with cognitive score when only demographic parameters were controlled, but this correlation was abolished for three PCs and six SMs after control of lipidemia, and for three SMs after further control of inflammatory variables. As the most abundant phospholipids in eukaryotic cells, PCs and SMs have positive influence on the incorporation of cholesterol in membranes [36]. In the LDL particle, approximately two thirds of the unesterified cholesterol is located in a mixed monolayer of PC and SM at the surface [37]. Additionally, several studies has reported higher concentrations of blood TC and LDL-C in late-life were associated with faster cognitive decline [38]. Thus, the variation of PCs and SMs that accompanies the decline in cognitive score might be a counter-balancing effect triggered by changes in cholesterol metabolism. Additionally, the confusing results on correlation between cognitive decline and PC/SM molecular species in previous studies might be due to the different covariate control schemes. Furthermore, although PS received interest as a dietary supplement for treatment of cognitive decline, few research presented association between circulating PS level and cognitive function [39]. Our results observed correlation between four PS molecular species and cognitive score for the first time according to our knowledge, but this relationship might be mediated by inflammatory similar with three SM molecular species.

Nevertheless, there are still several limitations of the protocol of the current study, which have to be taken into consideration. First, this study was of a cross-sectional design; therefore, no causal inferences could be made. Second, the participants included in this study were volunteered from three communities, but not randomly selected based on population, which might bring selection bias. Third, although we adjusted carefully for some covariates during the data analysis, residual confounding was still possible.

## 5. Conclusions

In conclusion, the association of plasma phospholipid profile with cognitive decline was investigated by characterization of 229 phospholipid molecular species in plasma samples from 626 senile residents. Significant association was confirmed between phospholipid matrix and MoCA score, and a total of 23 phospholipid molecular species were found to be associated with MoCA score independent of fasting glucose, lipidemia, lipoproteins, inflammatory variables and homocysteine. The decreased levels of pPEs containing LC-PUFA and the augmented levels of LPLs were the most prominent plasma phospholipid changes correlated with the cognitive decline, while alterations in plasma PC, PS and SM levels accompanying cognitive decline might be due to variation of lipidemia and inflammatory levels. Further studies, including more cohort studies or randomized clinical trials, are needed to confirm these observations.

## Figures and Tables

**Table 1:** General characteristics of study participants (*n* = 626).

	N (%) or Mean ± SD ^1^ or Median (IQR) ^2^
Sociodemographic variables	
Gender (male; *n*)	230 (36.7%)
Ethnicity (Han; *n*)	617 (98.6%)
Age (years)	64 (61–69)
Education level (*n*)	
≤6 years	145 (23.2%)
7–9 years	364 (58.1%)
>9 years	117 (18.7%)
MoCA score	25 (22–28)
Glu (mmol/L)	5.3 (4.9–6.0)
Lipidemia variables	
TC (mmol/L)	5.2 ± 0.9
TG (mmol/L)	1.2 (0.9–1.8)
LDL-C (mmol/L)	3.1 ± 0.9
HDL-C (mmol/L)	1.2 (1.0–1.4)
Lipoproteins	
Lp(a) (mg/L)	111.0 (54.5–265.4)
Apo A1 (g/L)	1.5 ± 0.3
Apo B (g/L)	0.9 ± 0.2
Apo E (mg/dL)	4.0 (3.2, 4.8)
Inflammatory variables	
CRP (mg/L)	0.9 (0.4, 1.8)
TNF-a (pmol/L)	10.2 (7.7, 14.5)
IL-6 (pg/mL)	102.5 (68.1, 145.7)
Hcy (μmol/L)	16.1 (13.0, 20.7)

^1^ SD: standard deviation; ^2^ IQR: interquartile range, expressed as lower quartiles (Q1)-upper quartiles (Q3).

**Table 2:** Concentration of phospholipid classes in plasma of participants.

	Molecular Species (*n*)	Concentration (mg/L)		
		Median	Q1 ^1^	Q3 ^2^
PC	62	1416.6	1155	1649.6
plasmanyl/plasmenyl PC	24	69.9	51.8	89.8
PE	55	42.9	33.5	54.7
plasmanyl/plasmenyl PE	39	7.3	5.9	8.9
SM	30	371.4	300.3	0.5
PS	22	1.6	1.3	2391.5
PI	19	35.3	27.1	46
PG	5	0.6	0.4	0.8
PA	3	0.3	0.2	2.3
LPC	22	135.4	99.3	464.6
LPE	11	4.1	3.2	175.3
Total	229	2046.7	1700.6	2391.7

^1^ Q1: lower quartiles; ^2^ Q3: upper quartiles.

**Table 3:** Distance-based linear model (DistLM) of phospholipid matrix with MoCA score and biochemical parameters ^1^.

	R^2^	SS_trace_	Pseudo-F	*p*
MoCA	0.054	92.173	4.278	<0.001
Glu	0.003	35.820	1.950	0.063
TC	0.066	931.140	46.375	<0.001
TG	0.027	374.060	19.177	<0.001
LDL-C	0.010	149.630	7.755	<0.001
HDL-C	0.004	52.088	2.707	0.019
Lp(a)	0.002	33.893	1.764	0.087
Apo A1	0.019	267.110	14.198	<0.001
Apo B	0.017	246.920	13.389	<0.001
Apo E	0.004	45.640	2.481	0.024
CRP	0.002	29.301	1.597	0.116
TNF-α	0.002	28.265	1.542	0.141
IL-6	0.001	17.332	0.945	0.422
Hcy	0.002	29.396	1.605	0.102

^1^ Gender, ethnicity, age and education level were forced into the model as control.

**Table 4:** Distribution of module eigengenes and their association with MoCA score ^1^.

Module	Module Eigengenes			Spearman’s Correlation	
	Median	Q1	Q3	*r_s_*	*p*
Blue	−0.001	−0.029	0.027	0.140	0.001
Cyan	−0.004	−0.028	0.025	0.047	0.239
Magenta	−0.003	−0.031	0.028	0.089	0.027
Salmon	0.000	−0.032	0.028	−0.010	0.796
Yellow	−0.006	−0.027	0.022	0.059	0.142
Midnightblue	−0.006	−0.029	0.027	−0.050	0.210
Green	−0.008	−0.024	0.010	0.047	0.240
Purple	−0.002	−0.028	0.028	0.024	0.548
Turquoise	−0.003	−0.030	0.029	0.056	0.166
Pink	0.001	−0.033	0.031	−0.075	0.060
Greenyellow	−0.001	−0.029	0.027	0.048	0.236

^1^ Gender, ethnicity, age, and education level were controlled in Spearman’s rank correlation.

**Table 5:** Correlation coefficients of Individual phospholipid molecular species associated with MoCA score ^1^.

Class	Molecular Species	Spearman’s Rank Correlation Coefficient				
		Model 1	Model 2	Model 3	Model 4	Model 5
PE	PE(P-46:8)	0.119 *	0.104 *	0.112 *	0.102 *	0.100 *
	PE(P-46:7)	0.149 *	0.137 *	0.130 *	0.115 *	0.114 *
	PE(P-44:8)	0.108 *	0.092 *	0.094 *	0.081 *	0.079 *
	PE(P-44:7)	0.135 *	0.117 *	0.122 *	0.107 *	0.107 *
	PE(P-44:6)	0.108 *	0.091 *	0.101 *	0.092 *	0.092 *
	PE(P-44:5)	0.099 *	0.086 *	0.086 *	0.072 *	0.072 *
	PE(P-42:8)	0.099 *	0.076	0.077	0.060	0.062
	PE(P-42:7)	0.108 *	0.086 *	0.086 *	0.071 *	0.073 *
	PE(P-42:6)	0.134 *	0.112 *	0.112 *	0.097 *	0.100 *
	PE(P-42:5)	0.125 *	0.102 *	0.103 *	0.093 *	0.095 *
	PE(P-42:4)	0.104 *	0.084 *	0.089 *	0.08 *	0.081 *
	PE(P-40:8)	0.082 *	0.074	0.075	0.061	0.060
	PE(P-40:7)	0.138 *	0.120 *	0.114 *	0.103 *	0.105 *
	PE(P-40:6)	0.119 *	0.095 *	0.088 *	0.075 *	0.079 *
	PE(P-40:5)	0.105 *	0.083 *	0.073	0.060	0.064
	PE(P-40:4)	0.093 *	0.066	0.064	0.044	0.048
	PE(P-38:6)	0.134 *	0.122 *	0.115 *	0.103 *	0.104 *
	PE(P-38:5)	0.109 *	0.084 *	0.075	0.060	0.064
	PE(O-40:4)	0.079 *	0.053	0.052	0.035	0.037
	PE(40:7)	0.082 *	0.079	0.076	0.066	0.068
	PE(40:6)	0.080 *	0.086 *	0.088 *	0.089 *	0.092 *
	PE(38:6)	0.090 *	0.089 *	0.093 *	0.093 *	0.095 *
PC	PC(O-32:0)	0.096 *	0.072	0.079	0.081	0.083
	PC(36:2)	0.079 *	0.057	0.059	0.068	0.068
	PC(35:3)	0.119 *	0.104 *	0.102 *	0.104 *	0.104 *
	PC(32:0)	0.079 *	0.047	0.055	0.056	0.058
SM	SM(d42:2)	0.097 *	0.073	0.076	0.074	0.073
	SM(d40:2)	0.097 *	0.068	0.067	0.063	0.064
	SM(d38:2)	0.105 *	0.076	0.076	0.080	0.081
	SM(d38:0)	0.082 *	0.056	0.051	0.050	0.052
	SM(d37:2)	0.084 *	0.059	0.051	0.049	0.051
	SM(d36:1)	0.143 *	0.126 *	0.117 *	0.113 *	0.114 *
	SM(d35:1)	0.116 *	0.099 *	0.090 *	0.076	0.078
	SM(d34:2)	0.100 *	0.077	0.072	0.054	0.054
	SM(d34:1)	0.104 *	0.087 *	0.088 *	0.076	0.076
	SM(d33:1)	0.113 *	0.094 *	0.081 *	0.057	0.060
PS	PS(36:5)	0.104 *	0.092 *	0.081 *	0.062	0.062
	PS(36:1)	0.096 *	0.090 *	0.082 *	0.079	0.081
	PS(34:3)	0.150 *	0.147 *	0.139 *	0.129 *	0.132 *
	PS(32:0)	0.096 *	0.097 *	0.089 *	0.068	0.068
PI	PI(35:2)	−0.087 *	−0.121 *	−0.110 *	−0.114 *	−0.114 *
LPE	LPE(16:0)	−0.103 *	−0.125 *	−0.116 *	−0.098 *	−0.098 *
LPC	LPC(24:0)	−0.098 *	−0.116 *	−0.106 *	−0.096 *	−0.095 *
	LPC(22:0)	−0.106 *	−0.121 *	−0.109 *	−0.098 *	−0.098 *
	LPC(16:1)	−0.094 *	−0.078 *	−0.086 *	−0.088 *	−0.087 *

^1^ Model 1: gender, ethnicity, age and education level controlled; Model 2: Model 1 plus Glu, TC, TG, LDL-C and HDL-C controlled; Model 3: Model 2 plus Lp(a), Apo A1, Apo B and Apo E; Model 4: Model 3 plus CRP, TNF-α and IL-6; Model 5: Model 4 plus Hcy. * *p* < 0.05, significance are labelled bold.

## Data Availability

Not applicable.

## Supplementary Materials

The following are available online at https://www.mdpi.com/article/10.3390/nu13072185/s1, Table S1: Standards and isotope internal standards used for quantification of phospholipid molecular species, Table S2: Quantitative ion information in MS/MS system, concentration distribution and module information in the WGCNA model of the identified phospholipid molecular species.
