# Supplementary material for: Association of Plasma Phospholipids with Age-Related Cognitive Impairment: Results from a Cross-Sectional Study
Source: Nutrients. 2021 Jun 25;13(7):2185. doi: 10.3390/nu13072185 (PMC8308406; doi:10.3390/nu13072185)
Supplement: Supplementary file 1 [file nutrients-13-02185-s001.zip › nutrients-1257853-supplementary.pdf]

Table S1 Standards and isotope internal standards used for quantification of phospholipid molecular species

Standard	Exact Mass	Precursor ion (m/z)	Product ion (m/z)	Internal standard
PC(14:0/14:0)	677.500	678.50	184.10	PC(15:0/18:1) (d7)
PC(15:0/18:1)	745.560	746.55	184.10	
PC(17:0/17:0)	761.593	763.60	184.10	
PC(18:0/18:0)	789.625	790.65	184.10	
PC(18:1/18:1)	785.593	786.60	184.10	
PC(18:2/18:2)	781.562	782.55	184.10	
PC(20:0/20:0)	845.687	846.70	184.10	
PC(22:0/22:0)	901.750	902.75	184.10	
PE(14:0/14:0)	635.453	636.45	495.45	PE(15:0/18:1) (d7)
PE(15:0/18:1)	730.520	704.50	563.50	
PE(17:0/17:0)	719.547	720.55	579.55	
PE(18:0/18:0)	747.578	748.60	607.60	
PE(18:1/18:1)	743.547	744.55	603.55	
PE(18:2/18:2)	739.515	740.50	599.50	
SM(d18:1/12:0)	646.505	647.50	184.10	SM(d18:1/18:1) (d9)
SM(d18:1/16:0)	702.568	703.60	184.10	
SM(d18:1/18:0)	730.599	731.60	184.10	
SM(d18:1/18:1)	728.583	729.60	184.10	
PS(14:0/14:0)	701.424	680.45	495.45	PS(15:0/18:1) (d7)
PS(16:0/16:0)	757.487	736.50	551.50	
PS(15:0/18:1)	769.490	748.50	563.50	
PS(18:0/18:0)	813.550	792.55	607.55	
PS(18:1/18:1)	809.518	788.55	603.55	
PI(16:0/16:0)	827.552	809.50	255.25	PI(15:0/18:1) (d7)
PI(15:0/18:1)	839.550	821.50	241.20	
PI(18:0/18:0)	883.615	865.60	283.30	
PI(18:1/18:1)	879.584	861.55	281.25	
PG(14:0/14:0)	688.429	665.45	227.20	PG(15:0/18:1) (d7)
PG(16:0/16:0)	744.492	721.50	255.25	
PG(15:0/18:1)	756.490	733.50	281.25	
PG(18:0/18:0)	800.554	777.60	283.30	
PG(18:1/18:1)	796.523	773.55	281.25	
PA(14:0/14:0)	614.392	591.40	227.20	PG(15:0/18:1) (d7)
PA(16:0/16:0)	670.455	647.45	255.25	
PA(18:0/18:0)	726.518	703.55	283.30	
PA(18:1/18:1)	722.486	699.50	281.25	
LPC(13:0)	453.286	454.30	184.10	LPC(18:1) (d7)
LPC(17:0)	509.348	510.35	184.10	
LPC(17:1)	507.332	508.35	184.10	
LPC(18:1)	521.394	522.35	184.10	

LPC(19:0)	537.379	538.35	184.10	
LPE(14:0)	425.254	426.25	285.25	LPE(18:1) (d7)
LPE(16:0)	453.286	454.30	313.25	
LPE(18:0)	481.317	482.30	341.30	
LPE(18:1)	479.300	480.30	339.30	

Table S2. Quantitative ions and concentration distribution of the identified phospholipid molecular species and their module information in the WGCNA model

No.	Molecular species	Precursor ion (m/z)	Product ion (m/z)	Concentration (μg/L)			WGCNA module
				Median	Q1	Q3	
1	PC(30:0)	706.55	184.1	1086.7	737.0	1656.2	Yellow
2	PC(32:0)	734.55	184.1	35191.8	27826.7	45835.4	Yellow
3	PC(32:1)	732.55	184.1	15048.7	10345.4	22456.0	Yellow
4	PC(32:2)	730.55	184.1	8160.4	5837.9	11119.2	Yellow
5	PC(34:1)	760.6	184.1	137431.4	101029.8	194661.1	Salmon
6	PC(34:1(OH))	776.6	184.1	992.8	680.2	1498.1	Midnightblue
7	PC(34:2)	758.55	184.1	349772.4	298808.1	414727.1	Purple
8	PC(34:2(OH))	774.55	184.1	6701.5	4917.1	9920.4	Midnightblue
9	PC(34:3)	756.55	184.1	9183.1	7226.0	11522.6	Yellow
10	PC(35:1)	774.6	184.1	6903.9	4697.8	10569.4	Midnightblue
11	PC(35:2)	772.6	184.1	6917.3	5129.8	9301.4	Salmon
12	PC(35:3)	770.55	184.1	2117.2	1565.9	2711.5	Gray
13	PC(36:1)	788.6	184.1	36216.8	28290.7	48735.5	Yellow
14	PC(36:2)	786.6	184.1	217994.0	178261.9	277021.1	Yellow
15	PC(36:3)	784.6	184.1	55789.3	39050.7	73993.4	Salmon
16	PC(36:4)	782.55	184.1	87893.4	74051.2	107731.3	Yellow
17	PC(36:4(OH))	798.55	184.1	2174.8	1762.6	2736.8	Gray
18	PC(36:5)	780.55	184.1	4018.6	2834.1	5805.2	Salmon
19	PC(37:2)	800.6	184.1	1601.8	1309.3	2000.1	Salmon
20	PC(37:3)	798.6	184.1	2165.5	1602.8	2989.7	Midnightblue
21	PC(37:4)	796.6	184.1	4699.3	3848.8	5756.4	Salmon
22	PC(38:2)	814.65	184.1	8839.0	5901.8	13272.8	Salmon
23	PC(38:3)	812.6	184.1	36200.6	29476.5	46319.9	Yellow
24	PC(38:4)	810.6	184.1	110073.0	89999.5	138398.6	Yellow
25	PC(38:5)	808.6	184.1	37945.8	26982.5	56939.2	Salmon
26	PC(38:6)	806.55	184.1	44471.8	35027.3	59046.3	Yellow
27	PC(40:4)	838.65	184.1	5560.2	3676.0	8189.7	Salmon
28	PC(40:5)	836.6	184.1	13187.4	9950.6	17347.0	Yellow
29	PC(40:6)	834.6	184.1	27596.8	19178.9	39169.6	Yellow
30	PC(40:7)	832.6	184.1	4899.8	3177.5	7620.5	Salmon
31	PC(40:8)	830.55	184.1	1221.9	875.7	1831.1	Salmon
32	PC(42:4)	866.65	184.1	259.5	172.8	427.3	Midnightblue
33	PC(42:5)	864.65	184.1	515.3	321.1	782.8	Salmon
34	PC(42:6)	862.65	184.1	581.5	369.4	841.1	Salmon
35	PC(42:7)	860.6	184.1	521.0	352.0	749.3	Salmon
36	PC(42:8)	858.6	184.1	544.7	353.2	906.4	Salmon
37	PC(42:9)	856.6	184.1	604.8	429.0	813.0	Salmon
38	PC(44:4)	894.7	184.1	99.7	64.1	150.8	Salmon
39	PC(O-32:0)	720.6	184.1	5783.7	4394.8	7722.1	Yellow
40	PC(P-34:1)	744.6	184.1	7308.5	5602.9	9606.3	Salmon

41	PC(P-34:2)	742.6	184.1	9887.7	6639.4	13348.8	Salmon
42	PC(P-36:2)	770.6	184.1	3987.7	3058.5	5401.1	Salmon
43	PC(P-36:3)	768.6	184.1	5471.5	3963.0	7371.8	Salmon
44	PC(P-36:4)	766.6	184.1	8548.2	5803.4	11782.8	Salmon
45	PC(P-38:4)	794.6	184.1	5083.2	4017.4	6624.7	Salmon
46	PC(P-38:5)	792.6	184.1	2511.4	1614.9	3680.2	Salmon
47	PC(P-38:6)	790.55	184.1	2301.1	1872.9	2724.8	Yellow
48	PC(P-40:4)	822.65	184.1	2114.6	1524.9	3168.0	Salmon
49	PC(P-40:5)	820.6	184.1	2792.7	2015.3	3779.2	Salmon
50	PC(P-40:6)	818.6	184.1	1044.8	740.0	1533.0	Salmon
51	PC(P-40:7)	816.6	184.1	770.8	614.1	1008.2	Yellow
52	PC(P-42:3)	852.7	184.1	910.1	605.0	1346.1	Salmon
53	PC(P-42:4)	850.65	184.1	1351.2	1032.5	1869.6	Salmon
54	PC(P-42:5)	848.65	184.1	1373.1	869.1	2153.9	Salmon
55	PC(P-42:6)	846.65	184.1	423.7	244.9	621.9	Salmon
56	PC(P-44:4)	878.7	184.1	1955.9	1198.4	2967.7	Salmon
57	PC(P-44:5)	876.7	184.1	2150.4	1405.9	3109.1	Salmon
58	PC(P-44:6)	874.65	184.1	277.8	197.3	434.8	Salmon
59	PC(P-44:7)	872.65	184.1	353.0	232.2	508.0	Salmon
60	PC(P-46:5)	904.7	184.1	412.9	265.0	630.1	Salmon
61	PC(P-46:6)	902.7	184.1	223.1	141.7	352.4	Salmon
62	PC(P-46:7)	900.7	184.1	289.9	184.9	448.0	Salmon
63	PE(34:1)	718.55	577.55	1316.5	921.6	1876.2	Greenyellow
64	PE(34:2)	716.5	575.5	3834.1	2708.2	5558.8	Greenyellow
65	PE(34:3)	714.5	573.5	186.4	130.8	269.7	Greenyellow
66	PE(36:1)	746.55	605.55	2495.8	1889.0	3399.9	Greenyellow
67	PE(36:2)	744.55	603.55	11298.1	8408.6	15021.2	Greenyellow
68	PE(36:3)	742.55	601.55	2734.3	2064.1	3801.6	Greenyellow
69	PE(36:4)	740.5	599.5	1230.5	888.0	1660.5	Magenta
70	PE(38:3)	770.55	629.55	2646.6	2010.2	3459.7	Magenta
71	PE(38:4)	768.55	627.55	3391.0	2409.3	4830.8	Magenta
72	PE(38:5)	766.55	625.55	1183.9	865.8	1622.8	Magenta
73	PE(38:6)	764.5	623.5	1693.7	1221.3	2468.5	Magenta
74	PE(38:7)	762.5	621.5	64.1	47.9	83.5	Magenta
75	PE(40:5)	794.55	653.55	490.6	334.3	727.9	Magenta
76	PE(40:6)	792.55	651.55	1247.1	849.9	1773.0	Magenta
77	PE(40:7)	790.55	649.55	273.7	203.1	370.3	Magenta
78	PE(40:8)	788.5	647.5	58.9	47.0	76.1	Magenta
79	PE(O-34:1)	704.55	563.55	194.6	153.8	249.8	Cyan
80	PE(O-34:2)	702.55	561.55	193.2	150.2	248.6	Cyan
81	PE(O-36:2)	730.6	589.6	322.7	249.8	423.9	Salmon
82	PE(O-36:3)	728.55	587.55	605.6	480.4	788.5	Cyan
83	PE(O-36:4)	726.55	585.55	249.2	185.3	335.2	Blue
84	PE(O-38:2)	758.6	617.6	91.8	74.7	112.5	Grey

85	PE(O-38:3)	756.6	615.6	169.9	131.1	219.4	Blue
86	PE(O-38:4)	754.6	613.6	283.2	211.4	387.7	Blue
87	PE(O-38:5)	752.55	611.55	505.4	395.2	661.8	Blue
88	PE(O-40:4)	782.6	641.6	65.8	54.4	80.8	Blue
89	PE(O-40:5)	780.6	639.6	140.6	108.2	177.8	Blue
90	PE(P-34:1)	702.55	561.55	191.6	154.1	250.9	Cyan
91	PE(P-34:2)	700.55	559.55	132.9	101.0	173.4	Cyan
92	PE(P-36:1)	730.6	589.6	329.1	257.8	433.6	Salmon
93	PE(P-36:2)	728.55	587.55	618.7	490.3	805.8	Cyan
94	PE(P-36:3)	726.55	585.55	952.1	727.3	1273.6	Blue
95	PE(P-36:4)	724.55	583.55	99.4	73.0	131.2	Blue
96	PE(P-36:5)	722.5	581.5	13.3	9.4	18.2	Gray
97	PE(P-38:4)	752.55	611.55	527.5	409.0	688.1	Blue
98	PE(P-38:5)	750.55	609.55	331.2	261.7	424.5	Blue
99	PE(P-38:6)	748.55	607.55	130.9	105.8	166.2	Gray
100	PE(P-40:4)	780.6	639.6	148.6	121.6	186.0	Blue
101	PE(P-40:5)	778.6	637.6	212.8	158.1	272.5	Blue
102	PE(P-40:6)	776.55	635.55	177.4	134.3	223.1	Blue
103	PE(P-40:7)	774.55	633.55	90.3	69.5	111.8	Grey
104	PE(P-40:8)	772.55	631.55	110.4	86.3	141.9	Magenta
105	PE(P-42:4)	808.6	667.6	21.3	16.2	28.7	Blue
106	PE(P-42:5)	806.6	665.6	28.9	22.9	36.7	Blue
107	PE(P-42:6)	804.6	663.6	20.0	15.7	25.6	Blue
108	PE(P-42:7)	802.6	661.6	12.0	9.8	14.8	Blue
109	PE(P-42:8)	800.55	659.55	15.1	11.9	19.0	Blue
110	PE(P-44:4)	836.65	695.65	6.2	4.6	7.9	Blue
111	PE(P-44:5)	834.65	693.65	8.6	6.4	11.6	Grey
112	PE(P-44:6)	832.6	691.6	10.2	7.4	13.9	Blue
113	PE(P-44:7)	830.6	689.6	7.5	5.8	9.5	Blue
114	PE(P-44:8)	828.6	687.6	5.6	4.4	7.1	Grey
115	PE(P-46:6)	860.65	719.65	1.3	0.7	2.2	Grey
116	PE(P-46:7)	858.65	717.65	2.6	1.7	3.8	Grey
117	PE(P-46:8)	856.6	715.6	2.5	1.8	3.4	Blue
118	SM(d32:1)	675.55	184.1	6092.8	4696.9	7802.9	Turquoise
119	SM(d32:2)	673.55	184.1	611.9	442.1	893.1	Turquoise
120	SM(d33:1)	689.55	184.1	2596.4	2052.9	3497.7	Turquoise
121	SM(d34:0)	705.6	184.1	12791.6	8682.1	20351.8	Turquoise
122	SM(d34:1)	703.6	184.1	121533.6	88167.5	165177.5	Turquoise
123	SM(d34:2)	701.55	184.1	14320.1	11379.8	19403.1	Grey
124	SM(d35:1)	717.6	184.1	2672.4	2160.6	3338.2	Turquoise
125	SM(d35:2)	715.6	184.1	464.0	330.7	684.8	Turquoise
126	SM(d36:1)	731.6	184.1	26369.1	20958.7	33564.5	Turquoise
127	SM(d36:2)	729.6	184.1	5484.5	3771.7	8323.3	Turquoise
128	SM(d36:3)	727.6	184.1	518.0	315.2	776.9	Turquoise

129	SM(d37:1)	745.6	184.1	669.5	492.7	878.6	Grey
130	SM(d37:2)	743.6	184.1	151.5	120.1	201.8	Turquoise
131	SM(d38:0)	761.65	184.1	866.1	718.7	1071.2	Turquoise
132	SM(d38:1)	759.65	184.1	9733.0	7252.9	14551.6	Turquoise
133	SM(d38:2)	757.6	184.1	4341.6	3621.2	5204.9	Turquoise
134	SM(d38:3)	755.6	184.1	210.3	152.1	295.0	Turquoise
135	SM(d39:1)	773.65	184.1	2553.6	1942.3	3526.4	Turquoise
136	SM(d39:2)	771.65	184.1	509.2	325.7	908.2	Turquoise
137	SM(d40:0)	789.7	184.1	2668.6	1689.6	4447.6	Turquoise
138	SM(d40:1)	787.65	184.1	28315.1	22564.7	34560.7	Grey
139	SM(d40:2)	785.65	184.1	17160.5	14441.9	20735.3	Grey
140	SM(d40:3)	783.65	184.1	1334.1	923.4	2027.8	Turquoise
141	SM(d40:4)	781.6	184.1	344.9	249.0	470.8	Grey
142	SM(d41:1)	801.7	184.1	8400.6	6047.1	13523.9	Turquoise
143	SM(d41:2)	799.65	184.1	5826.0	4662.2	7433.4	Turquoise
144	SM(d42:1)	815.7	184.1	16990.3	12893.5	24409.7	Turquoise
145	SM(d42:2)	813.7	184.1	43037.5	35286.5	52205.1	Turquoise
146	SM(d42:3)	811.65	184.1	20869.7	16893.9	25813.7	Grey
147	SM(d42:4)	809.65	184.1	3114.5	2258.0	4309.9	Turquoise
148	PS(32:0)	736.5	551.5	35.5	22.6	62.4	Grey
149	PS(34:0)	764.55	579.55	1.0	1.0	20.7	Green
150	PS(34:1)	762.55	577.5	16.5	1.0	29.3	Green
151	PS(34:3)	758.5	573.5	27.6	19.8	36.8	Gray
152	PS(36:0)	792.6	607.55	91.6	48.4	163.9	Grey
153	PS(36:1)	790.55	605.55	200.2	142.9	319.4	Green
154	PS(36:2)	788.55	603.55	94.7	69.3	130.2	Green
155	PS(36:4)	784.5	599.5	16.4	10.1	23.7	Gray
156	PS(36:5)	782.5	597.5	8.1	1.0	12.3	Gray
157	PS(38:2)	816.6	631.55	9.6	1.0	18.5	Green
158	PS(38:3)	814.55	629.55	96.2	68.9	132.6	Green
159	PS(38:4)	812.55	627.55	411.9	299.4	584.1	Green
160	PS(38:5)	810.55	625.5	23.4	15.1	35.6	Green
161	PS(38:6)	808.5	623.5	7.9	1.0	12.5	Green
162	PS(39:3)	828.6	643.55	12.4	8.4	17.0	Gray
163	PS(39:4)	826.55	641.55	48.1	31.5	63.3	Green
164	PS(39:5)	824.55	639.55	135.2	121.1	151.1	Gray
165	PS(40:3)	842.6	657.6	7.2	1.0	12.0	Green
166	PS(40:4)	840.6	655.55	80.1	52.5	117.7	Green
167	PS(40:5)	838.55	653.55	99.4	74.4	141.9	Green
168	PS(40:6)	836.55	651.55	130.4	87.2	195.4	Green
169	PS(40:7)	834.55	649.5	2.9	1.0	11.1	Green
170	PI(32:1)	807.5	255.25	68.5	40.5	105.9	Purple
171	PI(34:0)	837.55	255.25	57.4	41.2	74.2	Purple
172	PI(34:1)	835.55	255.25	648.3	489.6	879.4	Purple

173	PI(34:2)	833.5	255.25	1383.2	1034.5	1953.5	Purple
174	PI(35:2)	847.55	269.25	77.8	54.9	101.2	Purple
175	PI(36:1)	863.55	283.25	1351.5	1006.4	1819.2	Purple
176	PI(36:2)	861.55	281.25	6429.8	4706.0	8525.2	Purple
			283.25				
177	PI(36:3)	859.55	283.25	1148.9	863.8	1534.7	Purple
			281.25				
			255.25				
178	PI(36:4)	857.5	255.25	1444.0	1023.9	2050.2	Purple
179	PI(37:4)	871.55	269.25	110.4	82.1	147.3	Purple
180	PI(38:2)	889.6	283.25	231.6	164.4	308.3	Purple
181	PI(38:3)	887.55	283.25	3502.8	2696.1	4418.5	Purple
182	PI(38:4)	885.55	283.25	17248.7	13392.2	22563.0	Purple
183	PI(38:5)	883.55	283.25	58.5	41.9	81.2	Purple
184	PI(38:6)	881.5	255.25	149.0	106.8	209.4	Purple
			281.25				
185	PI(39:4)	899.55	297.3	53.4	39.7	72.5	Purple
			269.25				
186	PI(40:4)	913.6	311.3	178.5	136.1	234.2	Purple
			283.25				
187	PI(40:5)	911.55	283.25	399.1	299.6	549.2	Purple
188	PI(40:6)	909.55	283.25	505.4	366.4	681.8	Purple
189	PG(34:1)	747.5	281.25	378.3	276.7	497.5	Greenyellow
190	PG(34:2)	745.5	279.25	119.4	82.0	167.3	Greenyellow
191	PG(36:1)	775.55	281.25	42.9	30.8	62.7	Greenyellow
192	PG(36:2)	773.55	307.25	45.2	33.7	58.6	Greenyellow
			279.25				
			281.25				
193	PG(36:3)	771.5	279.25	13.4	11.1	16.2	Grey
194	PA(38:5)	721.5	303.25	224.0	150.8	326.9	Gray
			283.25				
			255.25				
195	PA(39:4)	737.5	303.25	53.2	21.8	91.0	Gray
196	PA(40:7)	745.5	327.25	15.7	2.5	32.6	Gray
197	LPC(14:0)	468.3	184.1	567.1	413.0	811.6	Grey
198	LPC(16:0)	496.35	184.1	50464.5	39457.6	64283.3	Pink
199	LPC(16:1)	494.35	184.1	819.1	675.4	1015.7	Grey
200	LPC(17:0)	510.35	184.1	610.5	491.3	764.1	Pink
201	LPC(18:0)	524.35	184.1	34561.6	23236.4	53430.6	Pink
202	LPC(18:1)	522.35	184.1	14163.1	10144.0	19575.6	Pink
203	LPC(18:2)	520.35	184.1	17245.8	12683.9	21751.2	Pink
204	LPC(18:3)	518.35	184.1	282.9	195.6	407.9	Pink
205	LPC(19:0)	538.4	184.1	181.1	126.5	255.0	Pink
206	LPC(20:0)	552.4	184.1	265.7	174.1	394.0	Pink

207	LPC(20:1)	550.4	184.1	342.5	206.7	542.5	Pink
208	LPC(20:2)	548.35	184.1	652.1	427.6	937.4	Pink
209	LPC(20:3)	546.35	184.1	2014.0	1408.3	2812.2	Pink
210	LPC(20:4)	544.35	184.1	6354.3	4374.5	8760.2	Pink
211	LPC(20:5)	542.35	184.1	248.2	143.6	450.5	Pink
212	LPC(22:0)	580.45	184.1	139.8	72.9	236.8	Pink
213	LPC(22:4)	572.35	184.1	193.0	123.8	276.8	Pink
214	LPC(22:5)	570.35	184.1	484.7	344.3	700.0	Pink
215	LPC(22:6)	568.35	184.1	1205.6	747.8	1892.1	Salmon
216	LPC(24:0)	608.45	184.1	201.0	118.0	324.7	Pink
217	LPC(P-16:0)	480.35	184.1	29.2	23.3	36.8	Gray
218	LPC(P-18:0)	508.4	184.1	152.1	92.4	227.9	Pink
219	LPE(16:0)	454.3	313.3	807.6	549.6	1133.6	Pink
220	LPE(18:0)	482.35	341.35	1400.3	1076.3	1887.9	Pink
221	LPE(18:1)	480.3	339.3	342.1	272.3	438.5	Greenyellow
222	LPE(18:2)	478.3	337.3	640.6	475.7	799.5	Grey
223	LPE(20:2)	506.35	365.35	11.9	8.4	17.5	Grey
224	LPE(20:3)	504.3	363.3	63.6	45.7	85.4	Pink
225	LPE(20:4)	502.3	361.3	453.9	318.0	644.1	Pink
226	LPE(22:4)	530.35	389.35	13.5	8.6	22.2	Pink
227	LPE(22:5)	528.3	387.3	62.4	40.5	95.0	Pink
228	LPE(22:6)	526.3	385.3	234.5	164.7	356.6	Pink
229	LPE(24:0)	566.4	425.4	5.9	4.2	8.9	Pink
